# Infection prevention and control measures for preterm infants discharged into the community: a scoping review protocol

**DOI:** 10.1186/s13643-023-02236-y

**Published:** 2023-05-08

**Authors:** Kathryn Carruthers, Dorothy Hannis, Jonathan Robinson, Alan Armstrong

**Affiliations:** School of Health and Life Sciences, Teesside University, Middlesbrough, UK

**Keywords:** Child health, Infection prevention and control, Neonatal, Parents, Premature infants

## Abstract

**Background:**

Infection prevention and control (IPC) is an evidence-based and practical approach to prevention of harm by infection (Infection prevention and control https://www.who.int/health-topics/infection-prevention-and-control#tab=tab_1). IPC recommendations targeted at community-acquired infection aim to prevent illness and subsequent hospital readmission. Cohesive guidance for parents of preterm infants has not been clearly established. The review objectives are to identify and map the global characteristics of IPC measures/recommendations for parents of preterm infants discharged home to the community.

**Methods:**

The scoping review will be conducted using the JBI methodological approach for scoping reviews and reported following the Preferred Reporting Items for Systematic Reviews and Meta-Analyses Scoping Review extension (PRISMA ScR) and the PRISMA extension for reporting literature searches in systematic reviews. Electronic databases will be searched and limited by publication year (2013-present day). Grey literature, reference lists and expert-provided sources will be searched against predetermined criteria. A minimum of two authors will independently screen evidence sources and chart evidence on a predetermined charting form. Sources including IPC measures, or recommendations for parents of preterm infants during discharge planning or in the community/home, will be permitted within inclusion criteria. Limits include human studies only and evidence from 2013-present day. Recommendations aimed at professional implementation will be excluded. A descriptive summary of findings will be presented, with diagrammatic and tabular representation.

**Discussion:**

Collated evidence will guide future research which will subsequently aim to develop policy and enhance clinical approaches.

**Systematic review registration:**

This review has been registered on the Open Science Framework (OSF) 4th May 2021, available at https://osf.io/9yhzk.

**Supplementary Information:**

The online version contains supplementary material available at 10.1186/s13643-023-02236-y.

## Background

The World Health Organization (WHO) defines infection prevention and control (IPC) as an “evidence-based” [1], “scientific approach and practical solution designed to prevent harm caused by infection” [2]. IPC draws upon the disciplines and evidence base of infectious diseases, epidemiology and healthcare system burdens [3]. Health organisations including the WHO [4, 5] and European Centre for Disease Prevention and Control [6, 7] have produced technical guidance, campaigns, and reports to prevent and manage infections such as COVID-19.

Approaches to IPC in healthcare settings include strategies such as hand hygiene, wearing personal protective equipment, social distancing, patient movement considerations (one-way systems, improved signage), isolation areas, respiratory hygiene measures, increased environmental cleaning, consideration of ventilation such as opening windows and offering remote consultations [8]. IPC public guidance has included hand hygiene education, social distancing, isolation, testing, use of face masks and restriction of movement. Less clear information has been provided to parents regarding post-discharge prevention of infection in preterm infants. Prevention of nosocomial infection in the neonatal unit has been widely studied [9-11]. Despite readmission risks, less is known about parent-implemented community measures. Although IPC measures are wide ranging and target a broad spectrum of avoidable infections, measures are particularly critical to prevent the transmission of community-acquired respiratory infections in preterm infants.

The WHO defines preterm as “babies born alive before 37 weeks of pregnancy are completed” [12]. The organisation estimates that globally, 15 million babies are born prematurely each year, and that prematurity is the leading cause of death in children < 5 years [12]. Literature reporting the medical, educational, and behavioural consequences and complications of prematurity is vast. Synthesised literature on the long-term consequences of prematurity reported impact on the pulmonary system (vascular and alveolar development, increased asthma risk and decreased lung function), renal system (kidney disease and interrupted nephrogenesis), cardiovascular system (cardiac and vascular insults, dysfunction, hypertension, ischemic heart disease, heart failure), central nervous system (autism, mood disorders, intellectual disability) and the endocrine system (diabetes, obesity, metabolic syndrome, osteoporosis) [13]. Wide-ranging economic consequences for healthcare systems in high-income countries, families and wider society must be recognised. Family consequences include caring responsibilities, cost implications of health goods/interventions, nutritional needs, domestic work and home repairs [14].

Systematically reviewed reports of quality improvement for bronchopulmonary dysplasia (BPD) identified BPD (formerly chronic lung disease) as the most common morbidity in premature infants [15]. A further systematic review concluded that the risk of severe respiratory syncytial virus (RSV) disease is substantially higher in infants with BPD, increasing the length of hospital stay and intensive care unit stay, duration of oxygen supplementation and mechanical ventilation compared to non-BPD infants [16]. RSV is a seasonal common respiratory virus and a leading cause of morbidity and hospitalisation in the paediatric population [17]. When comparing health resource utilisation among preterm and term infants hospitalised with RSV, a systematic review concluded that irrespective of gestation, preterm infants have poorer outcomes and greater utilisation of health resources than term infants [18].

A 2020 Vietnamese cohort study found that of the 193 preterm infants studied from birth to 24-month corrected age, 47% were readmitted at least once in the first year and 22% in year 2. All causes across the 2 years were due to respiratory infections (70%) followed by other infectious diseases (15%), echoing findings of prior studies in high-income countries [19]. Recommendations included information provision for parents regarding illnesses and preventative practices to reduce readmission rates post-discharge [19]. A 1-year, 2019, Austrian, observational study aimed to research the differences in infection number and severity between 72 preterm and 71 full-term infants [20]. Results showed significantly higher infection rates and severity in the preterm infants with factors impacting infections including the number of siblings, pregnancy duration and length of stay in hospital. Recommendations included post-discharge comprehensive care and parent information about increased infection risk and infection prevention measures. The authors concluded that prophylactic IPC measures should include vaccination of all family members who have contact with the infant, hand hygiene and avoidance of high-risk environments [20].

Five-hundred eighty-three Canadian participants were surveyed in 2021, regarding parental knowledge of RSV and other respiratory infections in preterm infants, concluding that parental knowledge of prophylaxis eligibility criteria is essential to aid infection prevention and management [21]. A neonatal network piece highlighted the need for validation of parental concern regarding RSV, predischarge parental education, prevention strategies listed on a prepared letter for the family and prophylaxis importance [22]. A transition-home programme, in the United States of America (USA), was evaluated in relation to rehospitalisation rates of preterm infants and concluded that preventative strategies must include the social, environmental and medical risk factors [23]. The home-health nurse role in RSV prevention in the USA is described as including caregiver education strategies regarding hand hygiene, visitor limitation, day-care attendance, smoking, awareness of signs and symptoms and prophylactic immunisation [24]. Stakeholder knowledge was sought by the protocol author K. C. Service users and neonatal unit staff reported vague information and recommendations given at the clinician’s discretion. Neonatal unit advice varied on frequency, content and the duration of measures recommended. Despite implications for mortality, disease burden and economic impacts, recommendations are not clearly or consistently presented. This review aims to assimilate existing heterogeneous literature sources and provide clarity regarding the characteristics of recommendations.

A preliminary search of MEDLINE (EBSCO host), PROSPERO and the Cochrane Database of Systematic Reviews was conducted, and no current or ongoing reviews on the topic were identified. This provides justification that there is appropriate evidence and significance to substantiate a scoping review on this topic. The review objective is to identify and map the characteristics (form, content, context and mode of delivery) of IPC measures and recommendations for parents of preterm infants discharged home to the community. The assimilation of evidence identified in this scoping review will inform future research recommendations. From the proposed research recommendations made through this scoping review, further research around policy and practice to mitigate the risk of infection and re-hospitalisation would be possible.

## Review questions

1. What IPC measures and recommendations are available for parents/caregivers of preterm infants during discharge or on discharge home to the community to mitigate the incidence of infection and readmission to hospital?

### Secondary questions

i) What is the range (year and location) of evidence that is available regarding the knowledge provision of IPC measures/recommendations?
ii) From the available evidence, what specific IPC measures/recommendations are documented (content)?
iii) What are the characteristics of the IPC measures/recommendations, including the form, source, mode of delivery and the context of provision of information to parents?

## Eligibility criteria

### Participants

This review will consider evidence that includes participants or sources (for example but not limited to healthcare professionals, government/third-sector organisation or peers) who provide information regarding IPC measures/recommendations to parents/caregivers of preterm infants or parents/caregivers of preterm infants who have received IPC measures/recommendations. Preterm infants may also be participants in studies that provide IPC measures/recommendations for parent/caregivers. A preterm infant will be defined as a baby born at < 37 weeks’ gestation [12].

### Concept

The core concept is parent/caregiver implemented IPC measures and recommendations and infection risk mitigation in the community. Eligible sources must provide recommendations and or risk mitigation strategies, with the aim of prevention of community-acquired infection in the preterm child. Common IPC measures and recommendations include hand hygiene, reduction in contact with others and environmental cleanliness. There will be no restriction on the background of the provider or the mode or form of delivery.

### Context

This review is not limited to provision of recommendations from a specific healthcare setting or organisation. The recommendations and measures to be included in this review may be recommended or provided prior to discharge of the preterm infant (for example during the discharge process, education classes or packages) or post-discharge but with intended implementation of such recommendations to be conducted within the home or community environment by the parent/caregiver. Evidence will be excluded if it pertains to implementation of measures in a healthcare setting, by a healthcare professional, or if the implementation is not intended to be provided by the parent/caregiver of the infant. Sources are not limited by geographical location.

### Types of sources

This scoping review will consider qualitative, quantitative and mixed-methods study designs and review pieces, for example systematic reviews. Evidence sources will be inclusive of grey literature and may include but are not limited to primary research studies, opinion pieces, conference abstracts, pamphlets, websites or blogs. Book chapters, dissertations and theses will be excluded from the review. The review will include sources of evidence from 2013-present day to capture recent innovations in neonatal care. Sources must be either written in the English language or have a translation available. Sources excluded by language will be recorded within the audit trail and reported to uphold transparency.

## Methods/design

The proposed scoping review will be conducted in accordance with the JBI Methodology for scoping reviews [25] and written using the JBI System for Unified Management, Assessment and Review of Information (SUMARI) [26, 27]. The search strategy and review will be reported in accordance with the Preferred Reporting Items for Systematic Reviews and Meta-analyses (PRISMA) extension for scoping reviews (PRISMA-ScR) [28] and the PRISMA-S extension to the PRISMA statement for reporting literature searches in systematic reviews [29].

### Search strategy

A peer-reviewed three-step search strategy will be used [30], aiming to locate all eligible evidence sources.

1. An initial limited search of MEDLINE (EBSCO host) and CINAHL was undertaken to identify the breadth and availability of literature on the topic. This preliminary search strategy contained key words for population, concept and context. The text words contained in the titles and abstracts of relevant articles, and the index terms used to describe the retrieved articles, were used to develop a full search strategy for MEDLINE with the assistance of an academic librarian (Table 1: Search strategy in Appendix). MeSH and key term variations were considered.
2. The search strategy, including all identified keywords and index terms, will be adapted for each included database and/or information source.
3. The reference list of all included sources of evidence will be screened for additional evidence sources (citation searching). Searches of grey literature will be conducted via databases and repositories. When required, the authors of the papers and experts in the field will be contacted for further information and to elicit knowledge of newly published sources. The search process will be iterative, and the search strategy may be modified to improve sensitivity and specificity. Any adaptations will be documented in an audit trail. Due to resource issues and translation feasibility, sources published in the English language or with an English language translation available will be included. Primary studies with an English language abstract will be included provided that appropriate information may be gathered. Studies excluded due to language will be recorded within the audit trail to uphold transparency. The search will be re-run prior to final analysis.

### Information sources

A comprehensive search of electronic databases will be conducted including MEDLINE (EBSCO), Embase (Ovid), CINAHL (EBSCO), PsycINFO (EBSCO), AMED (EBSCO), Cochrane Library Online, ProQuest Nursing and Allied Health Source, Directory of Open Access Journals, Science Direct, Scopus and Web of Science. Sources of unpublished studies/grey literature to be searched via online databases include OpenGrey, MedNar, Grey Literature Report, Health Management Information Centre and PsychEXTRA. Search engines Google (pages 1–20) and Google Scholar (pages 1–20) will also be used. Further potential grey literature sources will include, but will not be limited to, the National Centre for Health and Care Excellence (NICE), the Royal College of Paediatrics and Child Health (RCPCH), Royal College of Obstetricians and Gynaecologists and third-sector organisations and business stakeholders (for example BLISS and Tommy’s).

### Study/source of evidence selection

Following the search, all identified citations will be collated and uploaded into the EndNote 20 [31] citation management software by KC and duplicates removed. To mitigate the potential for disagreement, the following three-step pilot test framework will be followed:

1. Twenty-five titles and abstracts will be selected at random.
2. All reviewers will screen the 25 titles and abstracts using the eligibility criteria and stated definitions.
3. Formal evidence screening will commence when a minimum of 75% accuracy has been achieved [25]. An additional step of piloting the charting form will take place at this point (see data extraction).

Following this, the titles and abstracts will then be screened by a minimum of two independent reviewers for assessment against the review inclusion criteria for all the papers. Potentially relevant sources will be retrieved in full and their citation details imported into the JBI System for the Unified Management, Assessment and Review of Information (JBI SUMARI) (JBI, Adelaide, Australia) [26, 27]. The full text of selected citations will be assessed in detail against the inclusion criteria by two or more independent reviewers. If there is an unresolved disagreement following a discussion between reviewing authors, a third author will make the decision [25]. Reasons for exclusion of evidence sources at full text that do not meet the inclusion criteria will be recorded and reported. Search results and the study inclusion process will be reported in full in the final scoping review and presented in a PRISMA-ScR [32] flow diagram. The PRISMA-ScR and PRISMA-S checklists have been completed to ensure the methodological rigour of the protocol and have been submitted as supplementary files [29, 32].

### Data extraction

Data will be extracted by a minimum of two independent reviewers using a data charting tool (Table 2 in Appendix), with any disagreements resolved through discussion and/or an additional third reviewer. A draft charting form, adapted by the reviewers from the JBI template source of evidence details, characteristics and results extraction instrument [25], is provided (Table 2: Data extraction instrument in Appendix). The data extracted will include citation details, information regarding the participants, concept and context and key findings aligned to the review questions. The form was piloted by KC during the initial search and will be piloted by all reviewers in combination with the source of evidence selection piloting process. The form may be iteratively modified as necessary during the data extraction process. Any revisions will be clearly documented and detailed in the scoping review. If necessary, authors of papers will be contacted to request missing or additional data, where required.

### Data analysis and presentation

Data will be descriptively presented using a narrative summary. Frequency counts of concept characteristics will be tabulated [25]. Using tabular form, assimilated categories of recommendations (for example hand hygiene, restriction of crowded locations) will be mapped against source type (for example primary study, clinical policy, third-sector website) to highlight the content of recommendations. A table producing quantitative frequencies will map references against source type, categories of recommendations, delivery mode (for example pamphlet, formal education programme, website), context (for example to prevent a specific virus during winter months) and whether parent feedback was reported. This data may then be graphically represented. It is expected that data presentation will be refined and expanded as the nature of the material becomes known. The evidence summary and research gaps will be presented in diagrammatic form. Retrieved literature will be separated by year/publication date to acknowledge IPC measures implemented due to the COVID-19 pandemic.

### Supplementary Information

**Additional file 1.**

## Appendix

**Table 1:** Search strategy. Database: MEDLINE (EBSCO host)

No	Query	Results
S1	(MH "Infant, Premature + ")	63,941
S2	TI ((preterm or pre-term or prematur* or pre-matur* or preemie*) N5 (bab* or neonat*)) OR AB ((preterm or pre-term or prematur* or pre-matur* or preemie*) N5 (bab* or neonat*))	25,774
S3	S1 OR S2	78,177
S4	(MH "Patient Discharge")	38, 496
S5	TI (discharg* or community or home* or "first year") OR AB (discharg* or community or home* or "first year")	1,606,134
S6	S4 OR S5	1,612,652
S7	TI ((recommend* or guid* or best practice or advice or protocol or policy or "follow* up" or knowledge or (identif* N3 risk) or supervis* or support* or continu* or instruct* or demonstrat* or explain* or program* or interven* or strateg* or education or (health N3 promotion) or monitor* or (care N3 plan*) or visit*)) OR AB ((recommend* or guid* or best practice or advice or protocol or policy or "follow* up" or knowledge or (identif* N3 risk) or supervis* or support* or continu* or instruct* or demonstrat* or explain* or program* or interven* or strateg* or education or (health N3 promotion) or monitor* or (care N3 plan*) or visit*))	11,924,920
S8	(MH "Infection Control + ")	70,119
S9	TI ((prevent* or reduc* or minimi* or decreas* or eliminat*) N5 (respiratory or infection or virus* or hospital* or rehospital* or admission or readmission or RSV or morbidity or mortality or risk)) OR AB ((prevent* or reduc* or minimi* or decreas* or eliminat*) N5 (respiratory or infection or virus* or hospital* or rehospital* or admission or readmission or RSV or morbidity or mortality or risk))	729,755
S10	S8 OR S9	788,876
S11	S3 AND S6 AND S7 AND S10	719
	Limit 1st January 2013–1st March 2023	421
	Search performed: 1st March 2023	
	This strategy was peer reviewed by senior academic health librarians and experienced academic staff with knowledge and attention to the Peer Review of Electronic Search Strategies (PRESS) guideline statement [30]	

**Table 2:** Data extraction instrument

**Scoping review data charting form**		
Scoping review details	Scoping review title	Infection prevention and control measures and recommendations for preterm infants discharged into the community: a scoping review protocol
	Scoping review question	What infection and prevention and control measures and recommendations are available for parents/caregivers of preterm infants discharged home to the community?
	Scoping review sub-questions	i) What is the range and extent of evidence of the provision of infection prevention and control measures and recommendations provided to parents of preterm infants discharged home to the community? ii) What are the characteristics (form, content, context, and mode of delivery) of the infection prevention and control measures and recommendations?
Inclusion/exclusion criteria	Population/participants Population: provider Population: recipient Participants: sample size	If applicable, indicate the characteristics of the participants/population provider (for example healthcare professional, third-sector organisation). Where appropriate, provide demographic information about the preterm infants
		Indicate the characteristics of the recipient (for example parent/caregiver or preterm infant). Where appropriate, provide demographic information about the preterm infants
		Indicate sample size if applicable
	Concept Concept: purpose, aims and objectives	Indicate the purpose, aims and objectives of the evidence source (for example to prevent a specific virus during winter months)
		Indicate whether the parent/caregiver experience of the recommendations/measures was sought
	Context Context: when Context: mode Context: time	Indicate the context in which the measures and recommendations were provided (for example as part of a discharge planning education session)
		Indicate the mode of delivery of measures and recommendations (for example pamphlet, formal education programme, website)
		Indicate any time frames, frequency or duration if the recommendations/measures were provided as part of an intervention
	Country of origin	State the country of origin of the evidence source/study
	Types of evidence source	Indicate the type of evidence source (for example primary study, clinical policy, third-sector website)
Citation information	Authors	Indicate all authors (last name, first name/initial)
	Date	Indicate the date of publication
	Title	Indicate the full title and subtitle of the source of article or publication
	Journal title or website title/name	Indicate the title and subtitle of the journal, website or source of evidence
	Website URL	Indicate the website URL if applicable
	Last update date	Indicate the last update of the document or webpage if applicable
	Volume	Indicate the volume number of the journal or source if applicable
	Issue	Indicate the issue number of the journal or source if applicable
	Page numbers	Indicate the page numbers of the source if applicable
	Report number/version	Indicate the report number and/or version of the source if applicable
Findings and content	Content of measures/recommendations	Provide the content of all measures/recommendations, measures, of interventions and/or risk mitigation strategies. For example, frequent handwashing
	Form and mode of delivery of measures/recommendations	Provide the mode of delivery and form of all measures/recommendations. Include information such as how and in what form the information was communicated to parents and in what context
	Results/findings	Indicate the results/findings of the evidence source when applicable
	Additional relevant data	Indicate any further relevant information, for example outcome measures, study design, methodology and ethical approval if available
Study reference	List of references	Indicate any references of interest from the reference list of the included study (after full-text review)

## Abbreviations

IPC: Infection prevention and control
WHO: World Health Organization
BPD: Bronchopulmonary dysplasia
RSV: Respiratory syncytial virus
USA: United States of America
SUMARI: System for Unified Management, Assessment and Review of Information
PRISMA-ScR: Preferred Reporting Items for Systematic Reviews and Meta-analyses extension for Scoping Reviews
